# Mice expressing the autism-associated neuroligin-3 R451C variant exhibit increased mucus density and altered distributions of intestinal microbiota

**DOI:** 10.1093/ismejo/wraf037

**Published:** 2025-02-27

**Authors:** Madushani Herath, Joel C Bornstein, Elisa L Hill-Yardin, Ashley E Franks

**Affiliations:** Department of Anatomy and Physiology, University of Melbourne, Grattan Street, Parkville, Victoria 3010, Australia; Department of Pediatric Surgery, McGovern Medical School, The University of Texas Health Science Center at Houston, Fannin Street, Houston, Texas 77030, United States; Department of Anatomy and Physiology, University of Melbourne, Grattan Street, Parkville, Victoria 3010, Australia; Department of Anatomy and Physiology, University of Melbourne, Grattan Street, Parkville, Victoria 3010, Australia; School of Health and Biomedical Sciences, STEM College, RMIT University, 225-245 Clements Drive, Bundoora, Victoria 3083, Australia; Department of Microbiology, Anatomy Physiology and Pharmacology, School of Agriculture, Biomedicine and Environment, La Trobe University, Plenty Road, Bundoora, Victoria 3086, Australia

**Keywords:** autism spectrum disorder, neuroligin-3, microbial dysbiosis, mucus layer, spatial distribution of bacteria

## Abstract

The intestinal mucus layer protects the host from invading pathogens and is essential for maintaining a healthy mucosal microbial community. Alterations in the mucus layer and composition of mucus-residing microbiota in people diagnosed with autism may contribute to dysbiosis and gastrointestinal dysfunction. Although microbial dysbiosis based on sequencing data is frequently reported in autism, spatial profiling of microbes adjacent to the mucosa is needed to identify changes in bacterial subtypes in close contact with host tissues. Here, we analysed the spatial distribution of the mucin-2 protein using immunofluorescence as well as total bacteria, Bacteroidetes, Firmicutes phyla, and *Akkermansia muciniphila* using fluorescent *in situ* hybridization in mice expressing the autism-associated R451C variant in the *Neuroligin-3* gene. We show that the *Neuroligin-3* R451C variant increases mucus density adjacent to the distal ileal epithelium in mice. The relative density of total bacteria, Firmicutes, and *A. muciniphila* was increased whereas the density of Bacteroidetes was decreased closer to the epithelium in *Neuroligin-3^R451C^* mice. In summary, the autism-associated R451C variant in the *Neuroligin-3* gene increases mucus density adjacent to the epithelium and alters microbial spatial distribution in the mouse distal ileum.

The intestinal mucus layer demarcates the enteric microbiota from the interior of the body and serves as the sole source of energy for mucus-residing bacteria [1]. Individuals with autism are at high risk of hospitalization due to gastrointestinal (GI) dysfunction [2], but the cause is unknown. An altered abundance of mucolytic bacteria and GI dysfunction [3, 4] and changes in the ratio of Bacteroidetes and Firmicutes phyla in individuals with autism [5, 6] have been reported. Although microbial dysbiosis has been identified in autism patients using 16S rRNA sequencing, spatial localization patterns of bacterial communities within the intestine have not been reported.

Mice expressing the autism-associated R451C variant encoding Neuroligin-3 (*Nlgn3*), a membrane protein located at neuronal synapses, show GI dysfunction, microbial dysbiosis and reduced macrophage density in caecum [7, 8]. Expression of *Nlgn3* in most enteric neurons, glial cells [9], and enteroendocrine cells (Supplementary Fig. 1) suggests that NLGN3 plays an important role in neurally mediated gut function. The R541C variant reduces *Nlgn3* expression in enteric neurons [9] and could contribute to GI dysfunction observed in *Nlgn3*^R451C^ mice. Given that the enteric nervous system (ENS) is involved in maintaining the integrity of the mucus layer ([10], reviewed in Herath et al., 2020), alterations in the ENS potentially affect mucus properties. However, the impact of this variant on intestinal mucus density and the distribution of mucosal microbial communities has not been investigated.

Here, we investigated the effects of the *Nlgn3* R451C variant on mucus layer density and the mucosa-associated microbial community in the mouse ileum. We combined a mucus preservation method (methanol Carnoy’s fixation), mucus immunostaining (using MUC2C3) and fluorescent *in situ* hybridization to localize bacteria within the mucus layer of the mouse distal ileum. Using the MATLAB-based BacSpace image analysis platform [11], the spatial distribution of Bacteroidetes and Firmicutes phyla as well as the mucus-degrading species *Akkermansia muciniphila* adjacent to the mucosal surface in wild-type (WT) and *Nlgn3^R451C^* mice was compared (two-way ANOVA). Firstly, BacSpace identifies the epithelial boundary via the spatial gradient of the DAPI signal, both automatically and manually. The “Subtract debris” tool distinguishes and subtracts microbial fluorescence from auto-fluorescent objects like plant material and shed epithelial cells in mucus. BacSpace calculates the mucus layer density and bacterial density based on the immunofluorescence gradient perpendicular to the epithelium, averaging the fluorescence signal intensity along the epithelium and normalizing it by distance from the epithelium into the lumen (Supplementary Methods 1).

The relative mucus layer density was significantly higher within 0–20 μm from the epithelium in *Nlgn3^R451C^* mice compared to WT (*P* **<** .0001). In both WT and *Nlgn3^R451C^* mice, maximal mucus density was reached approximately 11 μm from the mucus–epithelial border (WT: 0.84 ± 0.3, *Nlgn3^R451C^* mice: 2.55 ± 0.2, *P* = .003, Fig. 1A1–A4).

**Figure 1:**
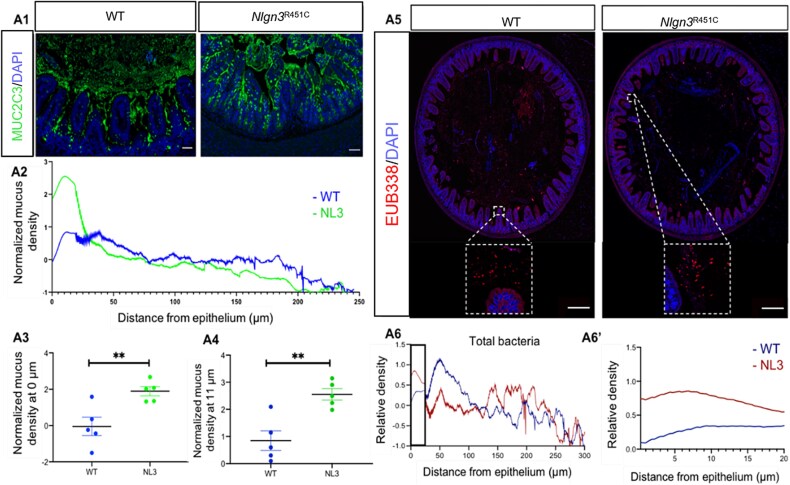
Ileal mucus layer density variation in the presence of the *Nlgn3* R451C variant. Confocal images of transverse sections of the distal ileum in (A1) WT and *Nlgn3*^R451C^ mutant mice. (A2) Mucus density variation from the mucosal to luminal direction averaged over the length of the epithelium in WT and *Nlgn3*^R451C^ mutant mice. (A3) Comparison of the average mucus density at the epithelium in WT and *Nlgn3*^R451C^ mice. (A4) The average mucus density comparison at 11 μm from the epithelium in WT and *Nlgn3*^R451C^ mice. (A5) Low-resolution confocal images of the distal ileum labelled with the universal bacterial marker EUB338, and DAPI, DNA marker in WT and *Nlgn3*^R451C^ mutant mice. Dashed insets: bacterial density in close proximity to the epithelium. (A6) Spatial distribution profile of total bacteria in WT and *Nlgn3*^R451C^ mutant mice. A6’ (enlarged area of interest indicated by a rectangle in (A6): Local distribution pattern of bacteria within 20 μm from the epithelium. Images captured using a Zeiss LSM800 confocal microscope, NA 0.8. Data are mean-subtracted and divided by the standard deviation for normalization (*n* = 5 in both groups). ^*^^*^P < .01, scale bar = 100 μm.

We compared total bacterial, phylum Bacteroidetes, phylum Firmicutes, and *A. muciniphila* density profiles from 0 to 20 μm from the epithelial border. The relative total bacterial density was increased in *Nlgn3^R451C^* mice compared to WT (*P* < .0001) (Fig. 1A5 and A6). *Nlgn3^R451C^* mice showed a lower density of Bacteroidetes 0–20 μm adjacent to the mucosa compared to WT (*P* < .0001).) (Fig. 2B1, B2). The density of Firmicutes within 20 μm of the epithelial boundary was higher in *Nlgn3^R451C^* mutant mice compared to WT (*P* < .0001) (Fig. 2B3, B4). The density of *A. muciniphila*, however, was significantly reduced close to the mucosa (within 10 μm of the mucosa), but increased above WT levels at a greater distance (i.e. further than 10 μm from the epithelium) in *Nlgn3^R451C^* mice (*P* < .0001) (Fig. 2B5, B6). Overall, bacterial density and localization patterns immediately adjacent to the mucosa (i.e. 0–20 μm from the mucosal barrier) were disturbed by the *Nlgn3* R451C variant.

**Figure 2:**
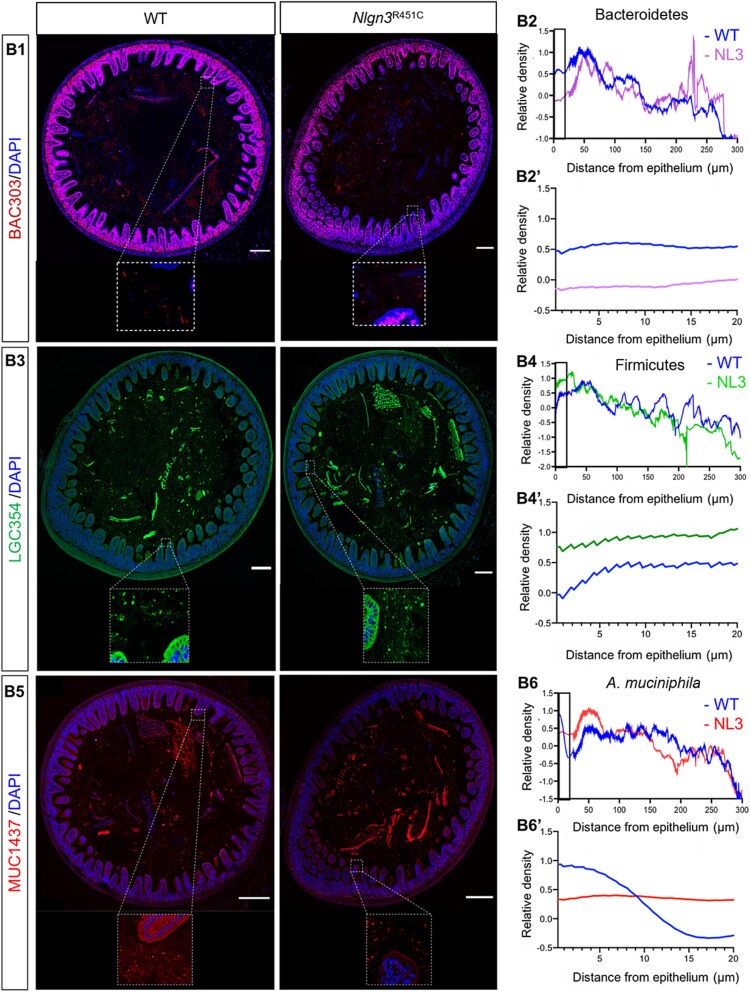
The R451C variant drives changes in bacterial localization in the distal ileum. (B1) Representative confocal images of distal ileum labelled with the BAC303 probe for the phylum Bacteroidetes^1^ and DAPI, a DNA marker for host nuclei in WT and *Nlgn3^R451C^* mutant mice. White inset: Bacterial density adjacent to the epithelium. (B2) Spatial distribution of Bacteroidetes (*n* = 5 WT and *n* = 5 *Nlgn3^R451C^* mice). (B2’) (enlarged area of interest indicated by black rectangle in B2): Bacteroidetes density within 20 μm from the epithelium. (B3) Confocal micrographs of distal ileum samples labelled with LGC354 FISH probe for the phylum Firmicutes^2^ and DAPI in WT (*n* = 3) and *Nlgn3*^R415C^ mice (*n* = 3). White insets: bacterial density adjacent to the epithelial boundary. (B4) Spatial distribution of Firmicutes. (B4’) (enlarged area of interest indicated by black rectangle in B4): The distribution of Firmicutes organization within 20 μm of the epithelial boundary. (B5) Confocal micrographs of the distal ileum labelled with MUC1437 probe for *a. muciniphila* and DAPI, DNA marker in (B5) WT and *Nlgn3^R451C^* mice. (B6) Spatial localization of *A. Muciniphila* in *n* = 4 WT and *n* = 5 *Nlgn3^R451C^* mice. (B6’) (enlarged area of interest indicated by black rectangle in B6): *Akkermansia muciniphila* Density from 20 μm adjacent to the epithelium. Images captured using a Zeiss LSM800 confocal microscope, NA 0.8. Data are mean-subtracted and divided by the standard deviation for normalization. Scale bar = 100 μm. After nomenclature changes, also known as ^1^Bacteroidota, ^2^Bacillota.

In the intestine, alterations in the mucosal microbiome are highly likely to occur together with changes in mucus layer properties because mucolytic bacteria such as *A. muciniphila* rely on mucus as the sole source of energy and nutrition. Because bacterial retention is commonly associated with increased mucus density [12], mucus accumulation may result in bacterial retention adjacent to the mucosa in the *Nlgn3^R451C^* mouse ileum. Although no previous studies have assessed the spatial distribution of bacteria in *Nlgn3^R451C^* mice, Earle and coworkers showed increased bacterial density within 20 μm adjacent to the distal colon mucosal epithelium in mice fed a diet deficient in microbiota-accessible carbohydrate compared to controls [11].

An imbalance in fecal Bacteroidetes and Firmicutes phyla was previously reported in autism [5, 13]. Similar to current findings in *Nlgn3^R451C^* mice, several studies reported lower Bacteroidetes:Firmicutes ratios in autism populations [6, 14, 15].

We show that *A. muciniphila* density is inversely correlated with mucus density in *Nlgn3^R451C^* mice. Pores in the mucus allow bacteria to penetrate the mucus layer and access carbohydrates, including O-glycan for use as nutrients and energy source [16]. Mucolytic species such as *A. muciniphila* exclusively feed on mucus O-glycans [17]; however, expansion of mucus structure is needed (regulated via pH and calcium levels, reviewed in Herath et al., 2020) for bacteria to penetrate and access tightly packed glycan chains. Impaired mucus expansion in *Nlgn3*^R451C^ mice might reduce accessibility to these O-glycans resulting in lower density of mucolytic bacteria.

Increased mucus density may lead to bacterial retention adjacent to the mucosa and impact overall microbial community structure and function in *Nlgn3*^R451C^ mice. Decreased abundance of Bacteroidetes alongside an increase in Firmicutes is associated with obesity and metabolic syndrome [18] and can lead to increased energy harvest from the diet and fat deposition*. A. muciniphila* also modulates the immune system, potentially reducing inflammation and improving immune responses as well as maintaining gut barrier function [19]. This study indicates that the *Nlgn3* R451C variant impacts mucus layer structure and composition; however, further work is necessary to elucidate the resulting microbial community level changes to function and biological mechanisms underlying neurally mediated mucus regulation in autism.

## Supplementary Material

Supplementary_figure-ISMEJ-D-24-00797_wraf037

## Data Availability

Data supporting this study are openly available at https://doi.org/10.1101/2022.06.27.497808
