# Supplementary material for: Mice expressing the autism-associated neuroligin-3 R451C variant exhibit increased mucus density and altered distributions of intestinal microbiota
Source: ISME J. 2025 Feb 27;19(1):wraf037. doi: 10.1093/ismejo/wraf037 (PMC11954627; doi:10.1093/ismejo/wraf037)
Supplement: Supplementary_figure-ISMEJ-D-24-00797_wraf037 [file supplementary_figure-ismej-d-24-00797_wraf037.pdf]

Supplementary figures

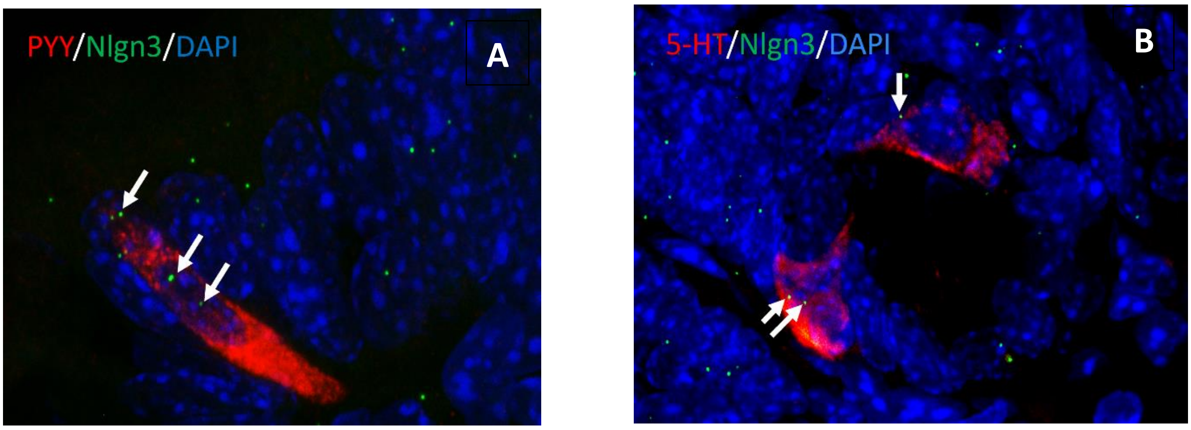

Supplementary figure 1: Expression of *Nlgn3* in (A) PYY-expressing enteroendocrine cells and (B) 5-HT expressing enterochromaffin cells in the adult WT mouse distal ileum indicating that low levels of Nlgn3 expression are observed in non-neuronal cells.

Supplementary methods

Selecting regions

To avoid bias, the full circumference of each cross section of distal ileal tissue was measured for bacterial localization. The ileal region of the GI tract was selected for analyses of bacterial localization based on previous data (Herath et al., 2022) showing that the *Nlgn3* R451C mutation impacts expression of *Nlgn3* in neurons and glia in the myenteric and submucosal plexus in the distal ileal region.

Distinguishing autofluorescence

BacSpace software eliminates autofluorescence from food particles. The ‘subtract debris’ tool of BacSpace will process each channel to remove debris using user-set thresholds. The user is able to adjust the first threshold to include as much debris as possible without including bacteria. After the primary thresholding, the secondary threshold can be used to identify additional debris.

Determining distance to epithelium

These distances were calculated using confocal images which were analysed in BacSpace software. In brief, an automated edge detection function within the BacSpace software detects both the interface between the epithelial-lumen surface and subsequently the interface between the lumen and the mucus border. This process is iteratively user-adjustable at high magnifcations, which enables highly accurate edge detection between the lumen and epithelial/mucosal surface for any selected image of an entire transverse section of the distal ileum, as utilised here. Within this process, there is a function enabling the user to identify and “subtract debris”. This enables any non-specific components (i.e. residual undigested plant-derived materials/autofluorescence) to be removed from the distance calculation.
Once this process has ascertained accurate estimations of the lumen-mucus and mucus-epithelial/mucosal boundaries, the distance is calculated based on a pixel-micron scale appropriate to the resolution of the captured confocal images of the tissue section (i.e. for this study the image resolution was 2048x2048 pixels, which was manually entered at the beginning of the analysis process during upload of the images).
